# Associations of Psychological Inflexibility with Exercise Self-Efficacy and Fatigue Severity among Individuals Seeking Treatment for Weight-Related Behaviors

**DOI:** 10.4148/2572-1836.1155

**Published:** 2022

**Authors:** Kara Manning, Brooke Y. Kauffman, Lorra Garey, Michael J. Zvolensky

**Affiliations:** University of Houston; University of Houston; University of Houston; Univiersy of Houston

**Keywords:** Fatigue, Obesity, Psychological Inflexibility, Exercise Self Efficacy

## Abstract

Rates of obesity are continuing to rise, contributing to several negative health outcomes and economic burden. Past work suggests that individuals with greater body mass index (BMI) are more likely to report feeling fatigue and are less likely to follow an exercise regimen, which may lead to weight-related problems. Psychological inflexibility, a rigid thinking style in which individuals attempt to over-control psychological reactions to discomfort, may be an underrecognized explanatory factor underlying greater fatigue and lower rates of exercise among individuals with weight-related concerns. The aim of the current study was to explore the relationship between psychological inflexibility and both exercise self-efficacy and fatigue severity among adults seeking treatment for weight-related behaviors. The current study is a secondary analysis and included 162 treatment-seeking adults who attended a baseline appointment for a larger randomized-controlled trial for weight-related behaviors. Results indicated that greater psychological inflexibility was significantly related to greater fatigue severity and lower exercise self-efficacy. These results provide initial empirical evidence that psychological inflexibility may be an important individual difference factor in terms of fatigue and exercise beliefs among adults seeking treatment for weight-related behaviors.

## Introduction

Obesity is a serious public health concern in the United States (CDC, 2020). Obesity is associated with numerous negative health outcomes, including diabetes, heart disease, stroke, and certain types of cancer (CDC, 2020). Indeed, obesity significantly shortens life expectancy (CDC, 2020) and is related to higher all-cause mortality (Global BMI Mortality Collaboration, 2016). Obesity is also associated with great financial burden in the United States, with annual medical costs alone averaging about $147 billion (CDC, 2020). Although nationwide health campaigns have been implemented to prevent and reduce obesity, prevalence rates have continued to increase (USDHHS, 2020). For example, over the past 20 years, obesity rates in the United States increased by 12%, such that approximately 42.4% of the population currently meet criteria for obesity (CDC, 2020). Thus, despite the well-established consequences of obesity, rates continue to rise in the general population and negatively impact health and mental health on a large scale (CDC, 2020).

Emerging work has identified that physiological factors play a critical role in the onset and maintenance of obesity (CDC, 2020). For example, fatigue, defined as an overwhelming sense of tiredness or weakness that can impact an individual’s ability to complete daily activities (Hockey, 2013; Junghaenel et al., 2011; Shen et al., 2006), may be an important construct to consider in the context of obesity. Individuals with obesity frequently report experiencing fatigue-related symptoms (Jarosz et al., 2014; Katz et al., 2016; Lim et al., 2005; Vgontzas et al., 2006) and fatigue symptoms are associated with greater body mass index (BMI; a measure of weight and obesity; Katz et al., 2016; Lim et al., 2005; Resnick et al., 2006; USDHHS, 2020; Vgontzas et al., 2006). Additionally, fatigue has been shown to result in an increase in weight-related problems (e.g., physical inactivity) among individuals with medical comorbidities (e.g., multiple sclerosis; Ozdogar et al., 2022). These findings are concerning as physical activity (i.e., the contraction of skeletal muscle that results in bodily movement that substantially increases energy expenditure; CDC, 2021b) and exercise (i.e., a subcategory of physical activity aimed to improve physical fitness through purposeful, planned, structured, and repetitive physical activity; CDC, 2021b) are important factors in maintaining a healthy BMI (Niemiro et al., 2019). Indeed, among individuals with obesity, exercise is associated with greater weight loss and is implicated in helping reduce food intake (Okay et al., 2009; Shaw et al., 2006; Wiklund, 2016). Extant work has found that exercise self-efficacy, beliefs about one’s ability to follow through with an exercise regimen in spite of deterring factors (e.g., mood, weather), may be an important aspect in initiating and maintaining an exercise regimen (Buckley, 2016; Linde et al., 2006; Napolitano et al., 2008). Thus, in addition to understanding contributing factors of fatigue symptoms and severity, understanding factors that may impact exercise self-efficacy is warranted.

Cognitive processing (e.g., attention, perception, and interpretation of the surrounding cues; Kreutzer et al., 2011) and response to physiological experiences (e.g., fatigue, exercise) may be critical to appreciate how our thinking leads to risk factors for obesity. Within this area of research, psychological inflexibility reflects a rigid thinking style in which individuals attempt to over-control psychological reactions to discomfort, decreasing an individual’s likelihood of living a value-driven life (Bond et al., 2011; Morton et al., 2020). For example, an individual with greater psychological inflexibility may believe that having painful memories will hold them back in life (Bond et al., 2011). Past work suggests that psychological inflexibility is related to poor mental health outcomes (Levin et al., 2014; Morton et al., 2020), as well as worse outcomes for individuals with chronic health conditions (i.e. greater levels of pain, worse disease maintenance, poorer treatment adherence; Hadlandsmyth et al., 2013; Talaei‐Khoei et al., 2017). Among individuals with chronic fatigue, lower psychological inflexibility has been shown to improve fatigue symptoms and decrease disability (Jonsjö et al., 2019; Novakov, 2021). Additionally, psychological inflexibility has been shown to be related to physical inactivity (Clemens et al., 2020), and decreasing psychological inflexibility may serve to improve physical activity adherence among individuals who struggle to maintain physical activity (Jenkins et al., 2019). Yet, available work on psychological inflexibility in the context of exercise, including beliefs and cognitions that impact exercise (i.e., exercise self-efficacy), is highly limited.

Theoretically, individuals with greater psychological inflexibility may be more likely to align with maladaptive thinking patterns in response to symptoms of fatigue and exercise. For example, individuals with greater psychological inflexibility may be more apt to believe that their fatigue is too overpowering to engage in adaptive behaviors (e.g., going for a walk, getting out of bed; Jonsjö et al., 2019). Similarly, these individuals may be inflexible to alternative thinking styles related to engaging in exercise (e.g., my depression makes it very difficult for me to exercise; Clemens et al., 2020). As a result of psychological inflexibility and associated maladaptive thinking patterns, these individuals may experience greater fatigue severity and lower self-efficacy to engage in an exercise regimen. Over time, these symptoms are likely to be related to problematic weight-related behaviors (e.g., decreased exercise, increased sedentary behavior) and increased likelihood for obesity (Chin et al., 2016; Katz et al., 2016; Lim et al., 2005; Resnick et al., 2006; Vgontzas et al., 2006).

The aim of the current study was to explore the relationship between psychological inflexibility and both exercise self-efficacy and fatigue severity among adults who completed a baseline appointment for a larger randomized-controlled trial for affectively vulnerable individuals with obesity seeking treatment for weight-related behaviors (Kauffman et al., 2022). It was hypothesized that greater psychological inflexibility would relate to lower exercise self-efficacy and greater fatigue severity, over and above the variance accounted for by age (Hallal et al., 2012), BMI (Resnick et al., 2006; USDHHS, 2020; Williams & Wood, 2006), and anxiety sensitivity (Kauffman et al., 2021).

## Methods

### Participants

Participants included 162 treatment-seeking adults (61.1% female, *M*_*age*_ = 31.57, *SD* = 10.68) who attended a baseline appointment for a larger randomized-controlled trial for weight-related behaviors (i.e., behaviors that contribute to fluctuations in weight such as eating or exercise behaviors; see Kauffman et al., 2022). Participants were invited to complete the baseline assessment if they met the following inclusionary criteria via an online self-report pre-screener: (1) being 18 years of age or older, (2) meeting criteria for obesity as defined by a BMI of 30 or greater calculated based on participant’s self-reported height and weight, and (3) endorsement of elevated anxiety sensitivity defined as a score of 17 or greater on the Anxiety Sensitivity Index–3 (ASI–3; Allan et al., 2014; Taylor et al., 2007). Participants were not invited to complete the baseline assessment if they self-reported legal matters that would have interfered with the ability to participate in the study (i.e., anticipation of upcoming incarceration that would lead to inability to complete study appointments) or not being fluent in English. The majority of the sample identified as white (61.1%) followed by black/African American (19.1%), Asian (9.3%), and Native American/Alaska Native (1.2%). A total of 9.3% identified as ‘other’ race and 24.1% identified as Hispanic/Latino. Approximately 57.4% of the sample reported clinically significant fatigue (i.e. scoring a 5 or above on the Fatigue Severity Scale; Bakshi, 2003; Ferentinos et al., 2011).

### Measures

#### Demographics.

Self-report data on demographic variables were collected from participants to describe the sample, including participant age, gender, race, and ethnicity. Age was utilized as a covariate in the current study.

#### Body mass index (BMI).

Self-reported height and weight were obtained to calculate participant BMI utilizing the recommended formula from the World Health Organization (i.e., [weight (pounds)]/[height (inches)^2^ × 703). BMI was utilized as a covariate in the current study.

#### Anxiety sensitivity.

The 18-item Anxiety Sensitivity Index–3 (ASI-3; Taylor et al., 2007) was used as a measure of fears related to physical, cognitive, and social consequences of experiencing anxiety-related symptoms and sensations. Participants are asked to rate on a 5-point Likert-type scale from 0 (very little) to 4 (very much) the extent to which they are concerned with the possible consequences of their anxiety symptoms (e.g., “It scares me when my heart beats rapidly.”). The ASI–3 has demonstrated adequate construct and convergent validity as well as excellent internal consistency in past work (α = .91; Farris et al., 2015; Kemper et al., 2012), and was utilized as a covariate in the current study (α = .91).

#### Psychological inflexibility.

The Acceptance and Action Questionnaire–II (AAQ–II) is a 7-item self-report measure that was utilized to assess for psychological inflexibility and experiential avoidance (Bond et al., 2011). Participants are asked to rate on a 7-point Likert-type scale from 1 (never true) to 7 (always true) the extent to which they identify with each statement (e.g., “Emotions cause problems in my life”). The AAQ–II has demonstrated good internal consistency (α = .78-.88) and adequate convergent and discriminant validity in past work (Bond et al., 2011), and demonstrated excellent internal consistency in the present study (α = .93).

#### Exercise self-efficacy.

The 5-item Exercise Self-efficacy (ESE) self-report measure was utilized to assess for participant confidence in the ability to engage in an exercise regimen (Linde et al., 2006). Participants were asked to rate various questions regarding self-efficacy to engage in exercise (e.g., “follow your exercise plan when your exercise workout is not enjoyable”) from 0 (not at all confident) to 8 (extremely confident). The ESE measure has demonstrated good content and construct validity (Kroll et al., 2007), and excellent internal consistency in past work (α = .91; Linde et al., 2006) and in the current study (α = .87).

#### Fatigue severity.

The 9-item Fatigue Severity Scale (FSS) was utilized in the current study as a measure of fatigue severity (Krupp et al., 1989). Participants were asked to indicate which response best fits with each statement (e.g., “Fatigue is among my most disabling symptoms.”) from 1 (strongly disagree) to 7 (strongly agree). The FSS has demonstrated adequate convergent validity (Learmonth et al., 2013) and excellent internal consistency in past work (α = .94; Impellizzeri et al., 2013), as well as in the present study (α = .92).

### Procedures

The present study is a secondary analysis from a larger randomized-controlled trial (see Kauffman et al., 2022). Participants were recruited nationwide through online advertisements (e.g., Facebook, Twitter) and within the local community through flyers and physician referrals. Participants who were interested in participating completed an online pre-screen survey. Potentially eligible participants were then invited to complete a baseline assessment in which an assessment of physical (e.g., pain, medical conditions) and mental health (e.g. experiential avoidance, anxiety sensitivity) constructs were administered. Inclusion and exclusion criteria were also re-assessed at the baseline assessment (see Kauffman et al., 2022). Participants who were not deemed eligible at the baseline assessment were compensated $10 and were dismissed from the study. Participants deemed eligible were invited to participate in an online intervention and follow-up assessments. The present study utilized baseline data collected as part of the larger trial. The current study was approved by the Institutional Review Board where the study took place.

### Data Analysis

SPSS version 28.0 was used to conduct the current analyses (2021). First, descriptive and zero-order correlations were examined among study variables. Then, two separate two-step hierarchical regression analyses were conducted for the following continuous criterion variables: (1) exercise self-efficacy and (2) fatigue severity. In the first step age, BMI, and anxiety sensitivity were added to the model. In the second step psychological inflexibility was added to the model. For each model, the *F* statistic was utilized to measure model fit and squared semi-partial correlations (*sr*^*2*^) were utilized as an indicator of effect size (interpreted as .01 = small, .09 = moderate, and .25 = large; Cohen, 1988). Statistical significance was set at *p* ≤ .05.

## Results

Zero-order correlations are presented in Table 1. Psychological inflexibility was significantly negatively related to exercise self-efficacy and positively associated with anxiety sensitivity and fatigue severity. Fatigue severity was statistically significantly and positively related to anxiety sensitivity and statistically significantly and negatively related to exercise self-efficacy.

Hierarchical regression results are presented in Table 2. For exercise self-efficacy, step 1 of the model with age, BMI, and anxiety sensitivity was not statistically significant (*R*^2^ = .02, *F*(3, 158) = 1.08, *p* = .358). In step 2 with psychological inflexibility added to the model, the model became statistically significant (*R*^2^ = .13, *F*(4, 157) = 5.62, *p* < .001) and accounted for a statistically significant increase in variance (Δ*R*^2^ = .11, *F*(1, 157) = 18.87, *p* < .001). Age and psychological inflexibility were statistically significantly associated with exercise self-efficacy.

In terms of fatigue severity, with covariates in the model for step 1, the model was statistically significant (*R*^2^ = .08, *F*(3, 158) = 4.31, *p* = .006); anxiety sensitivity was statistically significantly associated. The model with psychological inflexibility added in step 2 remained significant (*R*^2^ = .19, *F*(4, 157) = 9.06, *p* < .001). The addition of psychological inflexibility accounted for a statistically significant increase in variance (Δ*R*^2^ = .11, *F*(1, 157) = 21.61, *p* < .001). BMI and psychological inflexibility were statistically significantly related to fatigue severity.

## Discussion

The purpose of the current study was to evaluate the relationship between psychological inflexibility and both exercise self-efficacy and fatigue severity among adults seeking treatment for weight-related behaviors. Results regarding our *a priori* hypothesis were consistent with prediction. Specifically, psychological inflexibility significantly related to lower exercise self-efficacy and greater fatigue severity. These results were significant over and above the variance accounted for by age, BMI, and anxiety sensitivity. The size of the incremental effects of psychological inflexibility were moderate for exercise self-efficacy (11% of variance) and fatigue severity (11% of variance). These results are the first to empirically establish psychological inflexibility as a relevant factor for better understanding fatigue and exercise beliefs among adults seeking treatment for weight-related behaviors. Drawing from the current results, it may be theorized that individuals with greater psychological inflexibility may be particularly sensitive to uncomfortable mental or physical experiences (i.e., fatigue, depression, etc.), and thus may engage in maladaptive thinking patterns (i.e., “my depression makes it impossible to exercise”; Clemens et al., 2020). As such, these individuals may experience lower exercise self-efficacy and greater fatigue severity. Over time, such experiences may lead to problematic weight-behaviors (i.e., sedentary lifestyle), increasing the likelihood for obesity.

Although not a primary aim, it is important to bring attention to the degree of fatigue severity observed in the current sample. Specifically, the mean level of fatigue severity observed was 4.94 (*SD* = 1.35). This score is comparable to clinical levels of fatigue observed in individuals with chronic health conditions, such as multiple sclerosis (*M* = 5.00; Bakshi, 2003; Ferentinos et al., 2011). This finding is noteworthy as it provides further evidence that individuals with weight-related concerns experience elevated levels of fatigue (Katz et al., 2016; Lim et al., 2005; Resnick et al., 2006). More work needs to be dedicated towards better understanding the co-occurrence of these health concerns, as this may be a particularly ‘at-risk’ group for greater mental and physical health problems.

There are some limitations to the current study that warrant comment. First, the majority of the sample identified as white (60.7%), which limits the generalizability of the current findings to other diverse groups. Second, the data used in the current study was cross-sectional, limiting the ability to establish temporal associations between the studied variables. Future work would benefit from employing a longitudinal approach in order to establish the directionality of the effects between psychological inflexibility and both exercise self-efficacy and fatigue severity. Third, the current study was conducted remotely and therefore measures utilized for the current study were self-report. Future work would benefit from utilizing a multi-method assessment approach, including the use of laboratory paradigms to assess for more objective measures of the constructs of interest. Finally, the individuals in the current study were adults seeking treatment for weight-related behaviors. As such, these individuals may not be representative of all individuals with obesity.

Overall, the present findings uniquely build upon past work on psychological inflexibility, suggesting that it is an important factor related to lower exercise self-efficacy and greater fatigue severity among adults seeking treatment for weight-related behaviors. Findings from the current study may provide insight into cognitive processes (i.e., psychological inflexibility) guiding behaviors associated with weight-related problems.

### Implications for Health Behavior Theory

Based upon the current findings, it would be beneficial for all health providers to assess for psychological inflexibility among individuals presenting to treatment with weight-related concerns. It is clear from past work that severe fatigue and lack of exercise contribute to the maintenance of obesity status (Katz et al., 2016; Lim et al., 2005; Resnick et al., 2006; Vgontzas et al., 2006), thus, specifically addressing underlying individual difference factors, such as psychological inflexibility, may eliminate such barriers to healthy lifestyle changes. Should such psychological barriers be present, tailored treatment modalities for this population could be usefully pursued. For example, Acceptance and Commitment Therapy (ACT) has been shown to be a particularly beneficial treatment modality for reducing psychological inflexibility and guiding individuals towards living a value-based life (Hayes et al., 2012). ACT has also demonstrated efficacy among individuals with weight-related concerns by helping them identify personal barriers hindering positive behavior change (Kasila et al., 2020), which may be particularly beneficial for individuals with comorbid concerns. Future work should examine whether targeting psychological inflexibility in a clinical capacity fosters positive behavior change.

## Discussion Question

The average level of fatigue observed in the current sample was comparable to what is observed in health-compromised populations (i.e. multiple sclerosis). What factors do you think contribute to such elevated levels of fatigue among individuals seeking treatment for weight-related behaviors?

## Figures and Tables

**Table 1:** Zero-correlations among Study Variables (N = 162)

Variable	*M* (*SD*)	1	2	3	4	5	6

1. Age	31.57 (10.68)	-					
2. BMI	37.39 (6.00)	.021	-				
3. Anxiety Sensitivity	41.36 (14.12)	−.119	−.080	-			
4. Psychological Inflexibility	29.32 (10.40)	−.151	−.114	.491**	−		
5. Exercise Self-efficacy	14.88 (8.67)	−.105	−.011	−.081	−.307**	-	
6. Fatigue Severity	4.95 (1.36)	.039	.101	.238*	.390**	−.423**	-

*Note*.

* *p* < .01

** *p* < .001

**Table 2:** Hierarchical Regression Results

Exercise Self-efficacy							

	b	SE	β	t	p	CI (l)	CI (u)

Age	−0.09	0.06	−0.12	−1.47	.145	−0.22	0.03
BMI	−0.02	0.11	−0.02	−0.20	.840	−0.25	0.20
Anxiety Sensitivity	−0.06	0.05	−0.10	−1.20	.230	−0.16	0.04
Age	−0.12	0.06	−0.15	−2.00	.047	−0.24	0.00
BMI	−0.06	0.11	−0.04	−0.59	.559	−0.28	0.15
Anxiety Sensitivity	0.05	0.05	0.08	0.96	.338	−0.05	0.15
Psychological Inflexibility	−0.31	0.07	−0.38	−4.34	< .001	−0.46	−0.17

Fatigue Severity							

	b	SE	β	t	p	CI (l)	CI (u)

Age	0.01	0.01	0.07	0.87	.384	−0.01	0.03
BMI	0.03	0.02	0.12	1.57	.119	−0.01	0.06
Anxiety Sensitivity	0.02	0.01	0.26	3.31	.001	0.01	0.04
Age	0.01	0.01	0.10	1.42	.159	−0.01	0.03
BMI	0.03	0.02	0.15	2.06	.041	0.00	0.07
Anxiety Sensitivity	0.01	0.01	0.07	0.87	.388	−0.01	0.02
Psychological Inflexibility	0.05	0.01	0.39	4.65	< .001	0.03	0.07

*Note. N* for analysis is 162.
